# Perampanel effectiveness in treating *ROGDI*-related Kohlschütter-Tönz syndrome: first reported case in China and literature review

**DOI:** 10.1186/s12920-023-01728-z

**Published:** 2023-11-16

**Authors:** Linxue Meng, Dishu Huang, Lingling Xie, Xiaojie Song, Hanyu Luo, Jianxiong Gui, Ran Ding, Xiaofang Zhang, Li Jiang

**Affiliations:** 1 Department of Neurology, Children’s Hospital of Chongqing Medical University, Chongqing, People’s Republic of China; 2 National Clinical Research Center for Child Health and Disorders, Chongqing, People’s Republic of China; 3 China International Science and Technology Cooperation Base of Child Development and Critical Disorders, Chongqing, People’s Republic of China; 4 Ministry of Education Key Laboratory of Child Development and Disorders, Chongqing, People’s Republic of China; 5 Chongqing Key Laboratory of Pediatrics, Chongqing, People’s Republic of China

**Keywords:** Kohlschütter-Tönz syndrome, *ROGDI* gene, Perampanel, Epilepsy, Developmental delay

## Abstract

**Purpose:**

This study reported the first case of Kohlschütter-Tönz syndrome (KTS) in China and reviewed the literature of the reported cases.

**Methods:**

This patient was registered at the Children’s Hospital of Chongqing Medical University. The patient’s symptoms and treatments were recorded in detail, and the patient was monitored for six years. We employed a combination of the following search terms and Boolean operators in our search strategy: Kohlschütter-Tönz syndrome, KTS, and *ROGDI*. These terms were carefully selected to capture a broad range of relevant publications in PubMed, Web of Science, WHO Global Health Library, and China National Knowledge Infrastructure, including synonyms, variations, and specific terms related to KTS. The pathogenicity of the variants was predicted using SpliceAI and MutationTaster, and the structures of the *ROGDI* mutations were constructed using I-TASSER.

**Results:**

This is the first case report of KTS in China. Our patient presented with epilepsy, global developmental delay, and amelogenesis imperfecta. A trio-WES revealed homozygous mutations in *ROGDI* (c.46-37_46-30del). The brain magnetic resonance imaging (MRI) and video electroencephalogram (VEEG) were normal. The efficacy of perampanel (PMP) in treating seizures and intellectual disability was apparent. Furthermore, 43 cases of *ROGDI*-related KTS were retrieved. 100% exhibited epilepsy, global developmental delay, and amelogenesis imperfecta. 17.2% received a diagnosis of attention deficit hyperactivity disorder (ADHD), and 3.4% were under suspicion of autism spectrum disorder (ASD). Language disorders were observed in all patients. Emotional disorders, notably self-harm behaviors (9.1%), were also reported.

**Conclusion:**

*ROGDI*-related KTS is a rare neurodegenerative disorder, characterized by three classic clinical manifestations: epilepsy, global developmental delay, and amelogenesis imperfecta. Moreover, patients could present comorbidities, including ADHD, ASD, emotional disorders, and language disorders. PMP may be a potential drug with relatively good efficacy, but long-term clinical trials are still needed.

**Supplementary Information:**

The online version contains supplementary material available at 10.1186/s12920-023-01728-z.

## What is known

- Kohlschütter-Tönz syndrome (KTS) is an autosomal recessive disorder that manifests with severe global developmental delay, early-onset intractable seizures, spasticity, and amelogenesis imperfecta, leading to the discoloration of both primary and secondary teeth (yellow or brown) (OMIM, 2022).
- Research has demonstrated that the primary genetic cause of KTS is linked to biallelic mutations in the *ROGDI* gene.
- The genotype-phenotype correlation of *ROGDI-*related KTS remains unclear.

## What is new

- In this study, we presented the first case of *ROGDI*-related KTS in the Chinese population, which added to the existing knowledge of this spectrum disorder. The intronic variant c.46-37_46-30del was detected and predicted to be pathogenic by causing loss of function of ROGDI.
- Clinicians may contemplate perampanel therapy for KTS patients with epilepsy as it could potentially decrease seizure frequency and enhance motor development.
- We speculate that there may be a link between KTS and comorbidities, therefore, we advocate for strengthening psychological interventions and adopting multidisciplinary management approaches for KTS patients to prevent accidental injury.

## Introduction

Kohlschütter-Tönz syndrome (KTS) is an autosomal recessive disorder, initially reported in a Swiss family by Kohlschütter in 1974 [1]. KTS is a rare disorder that manifests with severe global developmental delay, early-onset intractable seizures, spasticity, and amelogenesis imperfecta, leading to the discoloration of both primary and secondary teeth (yellow or brown) (OMIM, 2022). KTS is clinically heterogeneous, with varying ages of onset of epilepsy, diverse manifestations of seizures, and varied relationships between intellectual disability and seizures [2, 3]. To date, research has demonstrated that the primary genetic cause of KTS is linked to biallelic mutations in the *ROGDI* gene. The vast majority of KTS patients who had undergone genetic testing were found to have *ROGDI* mutations [4–6].

Owing to the rarity of KTS, there was relatively little treatment evidence provided by case reports. Currently, there is no specific treatment available for KTS. In addition, the disease's clinical phenotype is heterogeneous, which leads to delays in diagnosis. As a result, clinicians often relied on symptomatic treatment methods, including the administration of anti-seizure medications (ASMs). Regrettably, the prognosis for patients with KTS was typically unfavorable. KTS patients often experienced not only global developmental delay but also develop refractory epilepsy (RE) and even died early due to progressive mental deterioration, which undoubtedly causes heavy psychological and economic burdens for patients and their families [1].

To improve the management of children with KTS, this study presented the clinical and genetic features of the first reported KTS patient in China, a young Chinese girl with homozygous *ROGDI* mutations. We observed that the patient responded well to perampanel (PMP), which may serve as a useful reference for treating KTS-related epilepsy. Additionally, we compiled data on all previously reported patients with *ROGDI*-related KTS, including their genotypes, phenotypes, treatments, and prognosis. We also discussed and summarized the possible mechanisms of PMP effectiveness in treatment from the perspective of molecular mechanisms. This study aimed to facilitate early diagnosis and treatment of KTS, ultimately improving patient prognosis and reducing suffering.

## Materials and methods

### Patient

The patient was registered at the Children’s Hospital of Chongqing Medical University (CHCMU), the largest pediatric medical center in Southwest China. We obtained her detailed clinical and genetic information and performed six years of follow-up.

The diagnostic criteria for KTS have not been clearly established yet. However, the main genetic etiology has been identified as *ROGDI* mutations. The patient’s clinical features manifest three core symptoms of KTS: global developmental delay, early-onset intractable seizures, and amelogenesis imperfecta [5]. Therefore, the patient's diagnosis was based on both genetic test results and clinical symptoms.

### Next-generation sequencing (NGS)

Before performing the study, written informed consent was obtained from legal guardians. This study was approved by the CHCMU Ethics Committee. The targeted NGS was performed following previously reported experimental procedures [7]. The average sequencing depth of the WES was 123.810. The sequencing result was aligned to the Genome Reference Consortium Homo sapiens (human) genome assembly GRCh37 (GRCh37/hg19) and compared with the established human *ROGDI* sequence (NM_024589).

### Sanger sequencing

Sanger sequencing was performed to validate the variant identified by NGS and for segregation analysis. Amino acid sequence alignment was performed using Genedoc software (https://github.com/karlnicholas/GeneDoc) for conservative analysis. Then we used SpliceAI and MutationTaster to predict the pathogenicity of the variants [7].

### Quality control

The patient was evaluated by two psychiatrists, and at least one family member accompanied her to the clinic visit. Follow-up care was primarily conducted on an outpatient basis. A designated primary caregiver was requested to conduct the follow-up interviews. All clinical data were meticulously organized, cross-checked, and independently analyzed by two investigators.

### Data retrieval methodology

We conducted an extensive search to identify relevant cases of KTS in the Chinese population. Our search strategy involved utilizing multiple databases, including PubMed, Web of Science, WHO Global Health Library, and China National Knowledge Infrastructure. We employed a combination of search terms, including Kohlschütter-Tönz syndrome, KTS, and *ROGDI* to ensure a comprehensive and systematic retrieval of relevant literature. Additionally, we manually reviewed the reference lists of identified articles to identify any additional pertinent studies. The period of retrieval was up to June 2023. By conducting a meticulous review of the available literature and analyzing the clinical data from the case presented here, we aim to shed light on the underreported aspects of KTS within the Chinese population, ultimately filling a crucial knowledge gap in this field.

### Case report

This is a female from China. No obvious abnormality was found during her mother's gestation period. Due to a fetal heart rate of fewer than 120 beats per minute, her mother underwent a cesarean section delivery. The child was born with a birth weight of 2.87 kg. There is no history of seizures or intellectual disability in her family. The parents of this patient are not endogamy or consanguinity.

At the age of seven months, the child was admitted to our hospital’s neurology clinic due to developmental delay. She exhibited an inability to raise her head, laugh, or grab objects. Physical examination revealed decreased muscle tension of limbs and laxity of the Achilles tendon. Gesell Developmental Schedules (GDS) assessment showed her total developmental quotient (DQ) was only 38.8 points, indicating a developmental delay. Peabody's developmental motor scale (PDMS) also showed her fine motor and gross motor abilities were only equivalent to those of a two-month-old child. After conducting a magnetic resonance imaging (MRI) scan of the brain, no abnormalities were detected. No discharges were found through a video electroencephalogram (VEEG). Consequently, on the recommendation of the neurology clinic, she began rehabilitation training.

At the age of 10 months, the patient experienced her first unprovoked generalized seizure, characterized by cyanosis of the face and lips, lips smacking, eyes staring, loss of consciousness, and no convulsive limb movements. The seizure occurred without a fever and lasted for nearly one minute. The patient's VEEG revealed an increase in δ and θ waves of 3–4 Hz during wakefulness, along with clinical symptoms, leading to a diagnosis of epilepsy. Treatment was initiated with valproic acid (VPA) at a dose of 17 mg/kg/d, which initially proved effective. The seizures were well controlled at the beginning. The longest time of seizure-free was 7 months with a maintenance dose of 27 mg/kg/d.

Uniform yellow discoloration and carious changes in her teeth were observed at the age of one. Although the epilepsy was controlled to some extent, she exhibited significant developmental delays compared to her peers. Despite the aid of VPA and rehabilitation therapy, her mental and motor functions still progressed slowly. At 1.3 years of age, she could hold, chase, laugh, and sit with her back arched at that time. The PDMS showed her gross motor function was only equivalent to a 5-month-old infant, and her fine motor function was only equivalent to a 7-month-old infant. The GDS indicated a DQ of 47, showing that her motor function and cognition progressed more slowly than before. The patient is 7 years old now and continues to exhibit profound global developmental delays. Her comprehension is limited to basic instructions, and she is unable to perform self-care tasks such as independent eating or managing urinary and bowel functions. Throughout our thorough and ongoing monitoring, the patient has not displayed any comorbid conditions, including ADHD, or ASD, except for a language disorder.

The patient remained seizure-free until she reached 1.5 years of age. Subsequently, for the following four years, she began to experience recurrent seizures with a consistent pattern. These seizures were consistently triggered by fever following respiratory or digestive tract infections. Notably, there were no seizures reported after the fever subsided. In response to this recurring pattern, the patient was maintained on VPA as a treatment. At the age of five, the patient began experiencing afebrile convulsions once a week. These seizures were characterized by facial and labial cyanosis, staring eyes, or sometimes only peri-labial cyanosis. As a result, the patient's anti-seizure therapy was modified to include a combination of levetiracetam (LEV) at a dose of 20 mg/kg/d along with VPA. After the addition of LEV, the child's motor and communication functions reportedly improved. However, the frequency of seizures increased, occurring two or three times a night. During the VEEG, discharge was observed on the right side of the frontal lobe during sleep, with occasional discharge seen on the left side of the frontal lobe. As a result, at the age of six, the patient's treatment regimen was adjusted. PMP was introduced as part of her treatment plan, and concurrently, the dosage of VPA was gradually reduced as the child approached her sixth year. The initial and maintenance dose of PMP was 2 mg/n. The patient had been seizure-free for nearly seven months and showed significant improvement in gross motor skills after receiving PMP treatment. Currently, the patient is receiving a combination of PMP with a maintenance dose of 2 mg/n and LEV with a maintenance dose of 43 mg/kg/d for anti-seizure therapy. The efficacy of PMP in treating seizures and intellectual disability was apparent.

The MRI was normal. Mild regurgitation of the tricuspid and pulmonary valves was detected through a cardiac ultrasound. The immune-related examination did not mention any obvious immune abnormalities. Blood metabolism screening at 10 months of age indicated a possible diagnosis of citrullinemia, but subsequent examinations were normal. During the course of the disease, the patient's blood ammonia levels exhibited multiple instances of increase, occurring on five occasions, with the highest recorded level peaking at 79.4 μmol/L, the patient has been receiving L-carnitine supplementation for a duration of approximately 9 months as part of her treatment regimen to address specific metabolic needs. After repeated examinations, the blood ammonia returned to normal, so the L-carnitine was gradually stopped. The blood ammonia level remained stable without L-carnitine and the result of trio-WES did not indicate the causative gene for citrullinemia.

**Fig. 1:**
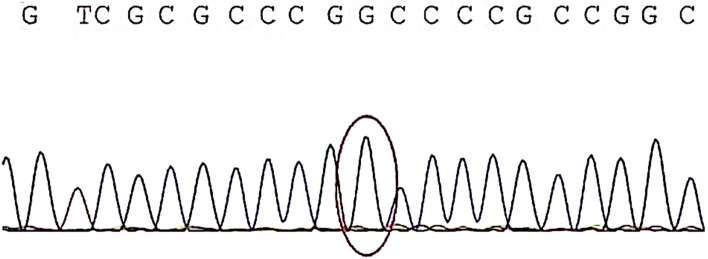
DNA sequence chromatogram of the *ROGDI* mutations. The circles indicate the position of the mutation. G: guanine; T: thymine; C: cytosine

**Fig. 2:**
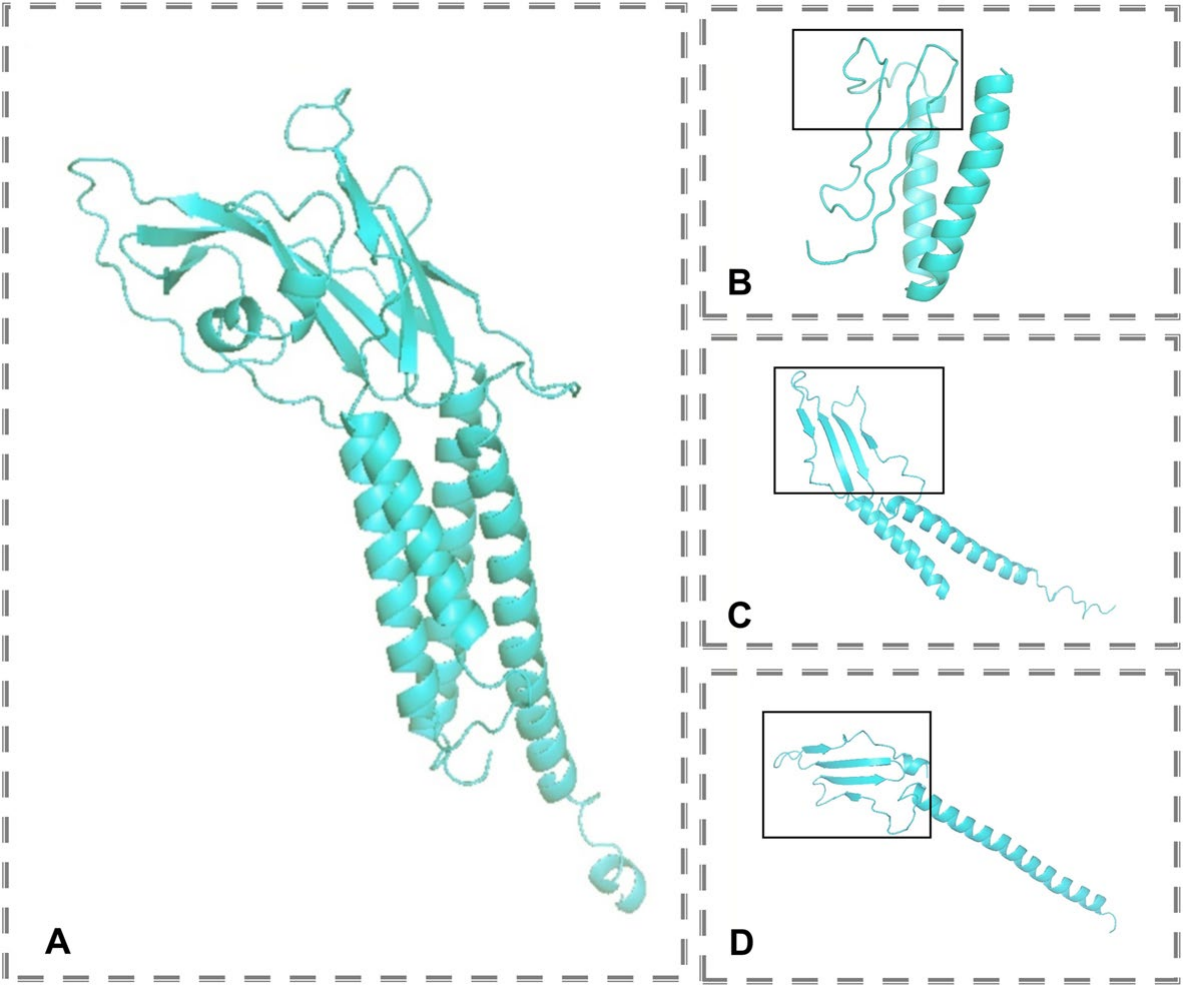
Molecular analysis of *ROGDI* missense variants. **A** Normal ROGDI protein structure. **B** c.286C > T/p. Gln96Ter, the peptide chain terminates at Gln96. **C** c.402C > G/p. Tyr134Ter, the peptide chain terminates at Tyr134. **D** c.469C > T/p. Arg157Ter, the peptide chain terminates at Arg157

When the patient was three years old, a trio whole exome sequencing (trio-WES) genetic examination was performed, which revealed homozygous mutations in *ROGDI* (c.46–37_46–30delGGCGGGGC) (Fig. 1). The patient's mother had heterozygous mutations at this site, while her father did not have any mutations at this site. Therefore, it is suspected that there may be a parental-single diploid on chromosome 16 in this patient. This variation was previously reported as a pathogenic mutation in a KTS patient. As shown in Fig. 2, The molecular structures resulting from nonsense variants in *ROGDI* were predicted by a hierarchical approach using I-TASSER. (https://seq2fun.dcmb.med.umich.edu//I-TASSER/).

In conclusion, the patient presented with the three core symptoms of KTS, including epilepsy, global developmental delay, and amelogenesis imperfecta. The patient's diagnosis of KTS was confirmed by the genetic examination results, which showed a homozygous deletion mutation of *ROGDI*. According to our follow-up, the patient has been seizure-free for seven months and meanwhile her gross motor skills had significant improvement after receiving a PMP treatment.

### Literature review

Forty-three cases of *ROGDI*-related KTS were retrieved. To comprehend the features of *ROGDI* gene mutations, we analyzed all KTS cases reported until June 2023 with pathogenic or likely pathogenic mutations of *ROGDI,* including the basic information (e.g., sex, age of onset, age of diagnosis), characteristics of seizures, comorbidities, and auxiliary examination. The neurologic manifestations of 44 KTS patients with pathogenic *ROGDI* variants were summarized in Table 1 (including our case) [1–5, 8–15]. We summarized a total of 44 KTS patients with *ROGDI* mutations (including our case), consisting of 25 (56.8%) males and 19 (43.2%) females. There was no clear chronological relationship between the onset of intellectual and developmental disabilities (IDDs) and epilepsy. Ten (22.7%) patients started with IDDs, and 34 (77.3%) patients started with epilepsy. The median age of seizure onset was 10 months (range: birth–48 months). It took an average of 37 months (range: birth–75 months) from seizure onset to establish the diagnosis of KTS. The seizure onset was triggered by fever in nine (20.5%) patients, and 11 (25.0%) patients had a history of febrile convulsions. The patterns of epilepsy were diverse, with six (40%) patients having generalized seizures, five (33.3%) patients having focal seizures, six (40%) patients having generalized tonic–clonic seizures, three (20%) patients having myoclonic seizures, one (6.7%) patient having unilateral tonic–clonic seizures, and one patient (6.7%) having generalized atonic seizures. Among those patients whose clinical information was available, three (6.8%) patients had status epilepticus, 24 (61.5%) patients developed RE, and three (7.7%) patients became seizure-free. Two (5.1%) patients had a history of encephalopathy. Descriptions of dystonia were reported in 34 patients, with 18 (52.9%) having dystonia and 16 (47.1%) not. Excluding one patient with incomplete documentation, 43 (100%) patients had IDDs. In those detailed cases reported, we also identified five (17.2%) patients with attention deficit hyperactivity disorder (ADHD) and one (3.4%) with suspected autism spectrum disorder (ASD). All 29 (100%) patients had language disorders, characterized by no or limited verbal language and communicated by shouting. Three (9.1%) patients had self-harm behaviors and showed more aggression than before, one (3.0%) had impulsiveness, and one (3.0%) showed a hyperactive temper. Based on the limited description, there is currently one case of hearing loss, and no visual impairment has been reported yet. There was no particular pattern of electrical discharge in the electroencephalogram (EEG). Of the 20 patients with VEEG or EEG descriptions, 17 (85%) patients had abnormal discharges. Brain MRI was described in 19 patients, of whom five (26.3%) were normal and 14 (73.7%) had diverse manifestations, including ventricular enlargement (28.6%), brain atrophy (35.7%), cortical atrophy (7.1%), delayed myelination (7.1%), marginally hypoplastic vermis (14.3%), corpus callosum agenesis (7.1%), and thickening of the right temporal lobe surface (7.1%). All the patients (100%) whose clinical data were available had amelogenesis imperfecta.

**Table 1:** The manifestations of 44 *ROGDI*-related KTS patients

Pt	Ethnic/Country	G	Initial symptoms	Age of diagnosis	Seizures					RE	Encephalopathy	Dystonia	IDDs	Behavior problem	Emotional impairment	Visual impairment	Amelogenesis imperfecta	EEG	MRI	Ref
					**Age of onset(m)**	**Trigger**	**Type**	**Febrile convulsion**	**Status epilepticus**											
1	Swiss	M	Seizure	NA	19	-	NA	-	-	NA	NA	+	+	NA	NA	NA	+	NA	NA	[1, 5]
2	Swiss	M	Seizure	NA	48	-	NA	-	-	NA	NA	NA	+	NA	NA	NA	+	NA	NA	
3	Swiss	M	Seizure	NA	21	-	NA	-	-	NA	NA	+	+	NA	NA	NA	+	NA	NA	
4	Swiss	M	Seizure	NA	18	-	NA	-	-	NA	NA	+	+	NA	NA	NA	+	NA	NA	
5	Swiss	M	Seizure	NA	11	-	NA	-	-	NA	NA	NA	+	NA	NA	NA	+	NA	NA	
6	Sicilian	M	Seizure	NA	11	-	NA	-	+	+	-	NA	+	NA	NA	NA	+	GE SLW	NA	[3, 9]
7	Sicilian	M	Seizure	NA	18	-	NA	-	-	+	-	NA	+	NA	NA	NA	+	NA	NA	
8	Sicilian	M	Seizure	NA	13	-	NA	-	-	+	-	NA	+	NA	NA	NA	+	NA	NA	
9	Sicilian	F	Seizure	NA	11	-	NA	-	-	+	-	NA	+	NA	NA	NA	+	NA	NA	
10	Sicilian	F	Seizure	48	22	-	NA	-	-	+	-	NA	+	NA	NA	NA	+	NA	NA	
11	Sicilian	M	Seizure	7	7	-	NA	-	-	+	-	NA	+	NA	NA	NA	+	NA	NA	
12	German	M	IDDs	NA	8.5	F	GS, FS	+	-	+	-	NA	+	ASD, LD	-	-	+	FO/MFO SSW	VE	[3, 15]
13	German	F	Seizure	NA	8	F	GS	+	-	+	-	NA	+	NA	-	-	+	GE SLW/FO SW	N	
14	Sicilian	M	Seizure	72	2	-	UTCSGTCSS	-	-	-	-	+	+	LD	-	-	+	MF SPW	BA	[3, 14]
15	Sicilian	F	IDDs	NA	10	F	MS	+	-	-	-	+	+	LD	-	-	+	SLW	VE	
16	Austrian	M	Seizure	NA	8	-	GA/GTCS	-	-	-	-	-	+	NA	-	-	+	FO/MF	BA, CH	[10]
17	Moroccan	M	Seizure	NA	4	F	GTCS	+	-	+	-	-	+	LD	-	L	+	MF	DM	[5]
18	Morocco	F	IDDs	NA	12	F	MS	+	-	+	-	-	+	LD	-	-	+	FO	DA	
19	German	M	Seizure	NA	11	F	NA	+	-	+	-	+	+	LD	-	-	+	NA	BA	[16]
20	Swiss	F	Seizure	NA	6	-	FS	-	-	SF	-	-	+	LD	-	-	+	FO	NA	[5]
21	German	M	Seizure	NA	7	-	NA	-	-	+	-	-	+	NA	-	-	+	NA	NA	[3]
22	Israeli	F	Seizure	NA	13	-	NA	-	-	-	-	+	+	LD	-	-	+	U	MHV	[4, 13]
23	Israeli	F	Seizure	NA	12	-	GS	-	-	+	-	+	+	LD	-	-	+	NA	BA	
24	Israeli	M	Seizure	NA	9	-	NA	-	-	-	-	+	+	LD	-	-	+	U	MHV	
25	Israeli	F	Seizure	NA	12	F	NA	+	-	-	-	-	+	LD	-	-	+	NA	NA	
26	Israeli	M	IDDs	NA	6.5	F	NA	+	-	+	-	+	+	LD	-	-	+	FO	N	
27	Israeli	M	Seizure	NA	42	-	NA	-	-	-	-	-	+	LD	-	-	+	MF	NA	
28	Israeli	F	IDDs	NA	9	-	NA	-	-	SF	-	-	+	LD	-	-	+	NA	NA	
29	Israeli	M	IDDs	NA	9	-	NA	-	-	+	-	-	+	LD	-	-	+	NA	NA	
30	Israeli	F	Seizure	NA	0	-	NA	-	-	+	-	-	NA	NA	-	-	NA	NA	NA	
31	Israeli	M	Seizure	NA	10	-	NA	-	-	-	-	+	+	LD	-	-	+	NA	NA	
32	Israeli	F	Seizure	NA	9	-	NA	-	-	+	-	-	+	LD	-	-	+	NA	NA	
33	Israeli	F-	Seizure	NA	9	-	NA	-	-	-	-	+	+	LD	-	-	+	NA	NA	
34	Israeli	M	Seizure	NA	9	-	NA	-	-	-	-	+	+	LD	-	-	+	MDD	NA	
35	Israeli	M	Seizure	NA	11	-	NA	-	-	+	-	-	+	LD	-	-	+	N	N	
36	Israeli	F	Seizure	NA	12	F	NA	+	-	-	-	-	+	ADHD, LD	HT	-	+	N	N	
37	Israeli	M	IDDs	NA	7	-	NA	-	-	+	-	+	+	LD	-	-	+	NA	NA	
38	Malian	F	IDDs	NA	0.75	-	GS, FS	-	+	+	+	+	+	LD	AG, IM	-	+	NA	NA	[11]
39	Indian	M	Seizure	NA	10	-	NA	-	-	-	-	-	+	ADHD, LD	-	-	+	NA	NA	[8]
40	Turkish	F	Seizure	2.2y	5	-	GTCS	-	-	SF	-	-	+	ADHD, LD	AG, SH	-	+	PISO	TRTLS	[2]
41	Turkish	F	IDDs	2.9y	7	-	GTCS, MS	-	-	+	-	+	+	ADHD, LD	AG, SH	-	+	N	BA, CCA	
42	Turkish	M	Seizure	6.8y	5	-	GTCS	-	-	+	-	+	+	ADHD, LD	-	-	+	NA	VE, CA	
43	Latvian	F	Seizure	6y	16	-	GS, FS	+	+	+	+	-	+	LD	SH	-	+	MF	VE	[12]
**44**	**China/Han**	**F**	**IDDs**	**3y**	**10**	**-**	**GS, FS**	** + **	**-**	** + **	**-**	** + **	** + **	**LD**	**-**	**-**	** + **	**SPW**	**N**	

*Pt* patient, *G* gender, *M* male, *F* female, *RE* refractory epilepsy, *IDDs* Intellectual and developmental disabilities, *NA* not available, *N* normal, *F* fever, *GS* generalised seizure, *FS* focal seizure, *VE* ventricular enlargement, *SSW* sharp slow waves, *FO* focal, *MFO* multifocal, *SLW* slow waves, *GE* generalized, *SW* sharp waves, *UTCS* unilateral tonic–clonic seizure, *GTCS* generalized tonic clonic seizure, *MS* myoclonic seizures, *SPW* spike waves, *BA* brain atrophy, *ASD* autism spectrum disorder, *GA* generalized atonic, *CH* cerebellar hypoplasia, *L* loss, *DM* delayed myelination, *MHV* marginally hypoplastic vermis, *SF* seizure free, *U* unremarkable, *MDD* mild diffuse discharge, *HT* hyperactive temper, *AG* aggressive, *IM* impulsive, *SH* self-harm, *PISO* paroxysmal irregularities of subcortical origin, *TRTLS* thickening of the right temporal lobe surface, *CCA* corpus callosum agenesis, *CA* cortical atrophy, *Ref* reference, *LD* language disorder. **Bold type**: Current case

**Fig. 3:**
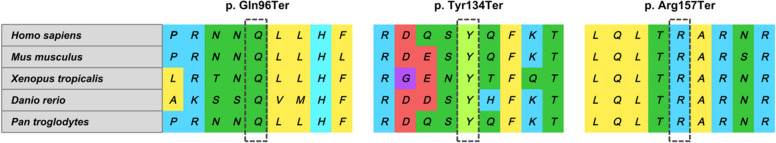
Conservation analysis of *ROGDI* nonsense variants. Conservation of the altered amino acid was shown in the MUSCLE alignment

To better comprehend the relationship between *ROGDI* genotypes and phenotypes, we summarized the genotypes of 44 patients in Table 2 and predicted their pathogenicity. Of the 44 patients, 15 (34.1%) had homozygous intronic variants, nine (20.1%) had homozygous frameshift mutations, 18 (40.9%) had homozygous nonsense mutations, one (2.3%) had heterozygous intronic mutations, and one (2.3%) had compound heterozygous mutations. It is noteworthy that all patients inherited mutations rather than de novo mutations. The *ROGDI* nonsense variants were located at extremely conserved positions (Fig. 3) All the nonsense mutations were predicted to be disease-causing by Mutation Taster. The protein structures resulting from nonsense mutations (Fig. 2) indicated a decrease in the effective ROGDI protein, which suggests the loss of ROGDI function. SpliceAI was used to predict the pathogenicity of intronic variants. All four intronic variants were predicted to affect splice sites, indicating their potential pathogenicity.

**Table 2:** Genotypes and protein function prediction of 44 *ROGDI*-related KTS patients

Pt	Position	Variant	Protein	Parental derivation	Prediction	
					Splice AI	Mutation Taster
1–5	-	c.531 + 5G > C	-	P	0.66	-
6–11	4,848,593	c.507del	p. Glu170 ArgfsTer72	P	-	-
12–13	4,852,507	c.45 + 9_ 45 + 20del	-	P	-	-
14–15	4,852,491	c.46-37_ 46-30del	-	P	-	-
16	4,850,549	c.286C > T	p. Gln96Ter	P	-	DC
17–18	4,851,292	c.229_230 del	p. Leu77 AlafsTer64	-	-	-
19	4,849,752 4,852,507	c.366dupA c.45 + 9_45 + 20del	p. Ala123SerfsTer19	F; M	-	-
20	4,848,565 4,848,187	c.531 + 5G > C c.532-2A > T	-; -	M; F	0.66 1	-; DC
21	4,848,593	c.507del	p. Glu170 ArgfsTer72	P	-	-
22–37	4,848,632	c.469C > T	p. Arg157Ter	P	-	DC
38	4,852,382	c.117 + 1G > T	-	P	0.99	DC
39	4,849,717	c.402C > G	p. Tyr134Ter	-	-	DC
40–42	4,851,323	c.201-1G > T	-	P	0.73	DC
43	4,851,268	c.255 + 1G > T	-	P	-	-
44	4,852,491	c.46-37_ 46-30del	-	M	-	-

*Pt* patient, *P* parents, *F* father, *M* mother, *DC* disease causing

To provide insights into the treatment strategies for KTS, we collected information on the treatment of 44 patients, and detailed descriptions of 15 of them are summarized in Table 3. Except for two patients who only used one anti-seizure medication (ASM), all other patients used at least two ASMs combined with antiepileptic therapy, and one patient used a maximum of five ASMs. None of the patients received adrenocorticotropic hormone (ACTH) treatment. One patient underwent vagus nerve stimulation (VNS) and adopted a ketogenic diet (KD), but this treatment did not have a satisfactory curative effect, and the patient still had RE. Seven patients (46.7%) responded well to ASMs, and six of them had a seizure frequency reduction of over 50%, with one becoming seizure-free at four years old. The seven patients responded well to different combinations of ASMs: one responded well to VPA combined with clobazam (CLB), one responded well to CLB, one responded well to phenobarbital (PHB), and vigabatrin (VGB), one responded well to CLB combined with phenytoin, one responded well to PHB combined with LEV, and two (including our case) responded well to PMP.

**Table 3:** Specific treatment and prognosis of 15 selected *ROGDI*-related KTS patients

Pt	ASMs		ACTH	VNS	KD	Prognosis	Controlling drug	Controlling age
	Num	Specific drugs (age)						
6	3	phenytoin, primidone, nitrazepam	-	-	-	LE	-	-
12	3	PHB, CBZ VPA	-	-	-	LE	-	-
13	2	PHB, CBZ	-	-	-	LE	-	-
14	4	PHB, CBZ, VPA, CLB	-	-	-	E	VPA, CLB	-
15	3	VPA, CBZ, CLB	-	-	-	E	CLB	-
16	2	PHB, VGB	-	-	-	E	PHB, VGB	-
17	1	LEV	-	-	-	LE	-	-
18	1	LEV	-	-	-	LE	-	-
38	5	CBZ, TPM, CZP, VGB, PHB	-	-	+	LE	-	-
39	2	Phenytoin, CLB	-	-	-	E	Phenytoin, CLB	-
40	2	PHB, LEV	-	-	-	E	PHB, LEV	4y
41	2	PHB, LEV	-	-	-	LE	-	-
42	3	CLB, ethosuximide, primidone	-	-	-	LE	-	-
43	2	VBZ, PMP	-	+	+	E	PMP	-
44	3	VPA, LEV, PMP	-	-	-	E	PMP	-

*ASMs* anti-seizure medications, *Num* number of types, *ACTH* adrenocorticotropic hormone, *LE* less effective, seizure frequency reduction less than 50%/ineffective/aggravated, *E* effective, seizure frequency reduction over 50%/seizure free, *PHB* phenobarbital, *KD* ketogenic diet, *CBZ* carbamazepine, *VPA* valproate, *CLB* clobacam, *VGB* vigabatrin, *LEV* levetiracetam, *TPM* topiramate, *CZP* clonazepam

## Discussion

The *ROGDI* gene consists of 11 exons and codes for a 287 amino acid protein. It is highly conserved across various species, mainly expressed in the human brain and spinal cord [16]. Prior to 2017, little was known about the protein encoded by *ROGDI*. However, the ROGDI protein is enriched at synaptic sites and co-localizes with the presynaptic scaffolding Bassoon protein, as well as the synaptic vesicle markers Synaptophysin, Synapsin-1, VAMP2/Synaptobrevin, and Mover [17]. *ROGDI* plays a crucial role in presynaptic targeting and facilitates efficient signal transmission.

*ROGDI*-related KTS has been reported in 43 cases, and the loss of function (LOF) has been proposed as a pathological mechanism [3]. Null mutations, including deletions, duplications, frameshifts, and intronic variations, have been identified in various cases of *ROGDI* mutations and may potentially lead to the complete LOF [5]. The c.507del deletion variant (located in exon 7) was found to be the genetic cause in 15.9% of KTS cases, leading to a frameshift mutation of *ROGDI* and LOF. Additionally, the c.229_230del deletion variant (located in exon 4) and the intronic variant c.531 + 5G > C (located in intron 7) were identified in 4.5% and 13.6% of KTS cases, respectively, and the latter was predicted to disrupt the splice donor site of *ROGDI*, both resulting in LOF. The c.45 + 9_45 + 20del intronic variant (located in intron 1) was identified in 6.8% of KTS cases, which might cause a splicing error that led to LOF of *ROGDI*. Furthermore, the nonsense variant c.469C > T (located in exon 7) was identified in 36.4% of KTS cases, predicted to cause premature termination of the peptide chain. The c.201-1G > T intronic variant (located in exon 3) was identified in 6.8% of KTS cases, predicted to have disrupted the splice acceptor site in exon 4, resulting in LOF of *ROGDI*. In this study, we also identified a homozygous variant (c.46–37_46–30del) in intron 1, predicted to have disrupted the splice acceptor site in exon 2, causing complete LOF of *ROGDI*. The pathogenicity of the homozygous variant has been confirmed in a previous KTS patient, leaving no room for doubt. One mutation site originated from the patient's asymptomatic heterozygous mother. It is speculated that the other mutation site also has maternal origin due to the exceedingly low probability of both alleles sharing the same mutation. This suggests a possible case of maternal uniparental disomy (mUPD) on chromosome 16. Uniparental disomy (UPD) is the inheritance of both copies of a chromosome or a chromosomal region from a single parent, as opposed to the typical inheritance of one copy from each parent. UPD occurs in two forms: paternal UPD (pUPD) and maternal UPD (mUPD), depending on whether the duplicated chromosomes originate from the father or mother. In cases of mUPD, individuals inherit both copies of a chromosome or chromosomal region exclusively from the mother, with no genetic contribution from the father, similar to the scenario in this case. mUPD can result from various mechanisms, including meiotic errors, non-disjunction events, and chromosomal rearrangements. Although our research did not entail a dedicated marker study, we advocate for future investigations to delve more comprehensively into this aspect. The rest variants listed in Table 2 were identified once. However, the number of cases is not sufficient for a possible mutational hot spot definition [2].

Epilepsy is the most common initial symptom in KTS patients. The age of seizure onset varies widely, with a median age of 10 months (range: 0–48 months). At present, no evidence indicates that early-onset seizures are a risk factor for a negative prognosis [1, 13, 14]. Seizure frequency typically decreases with age, expect individuals with RE [5, 13]. Our knowledge of KTS-related epilepsy is limited, and seizures can manifest in diverse forms without discernible patterns, as noted in prior studies [12, 14, 18]. Furthermore, the existing evidence does not adequately account for the relationship between genotype and seizure phenotype. This is exemplified by the observation that families with the same variation c.46–37_ 46–30del display diverse seizure patterns [14]. The assistance offered by VEEG/EEG results is also severely limited [5, 10, 12–14, 18]. Based on our summary of the VEEG/EEG results of the 44 KTS patients, we did not find any correlation between genotypes and discharge patterns. The exact pathogenesis of epilepsy in KTS patients caused by *ROGDI* gene mutations remains unknown. In view of the exocytosis of neurotransmitters and synaptic vesicle recycling are hallmarks of presynaptic function at mature synapses, Donatus Riemann et al. put forward one possibility that *ROGDI* may regulate exocytosis in neurons and the dysfunctional exocytosis would affect neural development and synaptic function [17]. Notably, *BASSOON*, which encodes the BASSOON protein and co-localizes with the ROGDI protein, has been recently identified as an epilepsy gene [19]. Additionally, *ROGDI* is involved in sleep regulation via dopaminergic signaling mediated by GABAergic pathways [20]. Therefore, *ROGDI* gene mutation might affect the expression of the ROGDI protein, affect the expression of the co-located BASSOON protein, and disrupt the *ROGDI*-GABAergic signaling pathway, ultimately leading to epilepsy. Nevertheless, this hypothesis needs further functional validation through experiments on animals or cells by silencing *ROGDI* expression and monitoring changes in *BASSOON* expression and the GABAergic signaling pathway.

Amelogenesis imperfecta is another significant symptom of KTS. To date, all KTS patients involve amelogenesis imperfecta. However, the underlying mechanism of *ROGDI*-related amelogenesis imperfecta remains unknown. KTS patients had very thin, soft, rough enamel and brown-stained enamel, which is susceptible to disintegration [1]. Yellow enamel was discovered when our patient was one year old. She was already displaying global developmental delay and epilepsy. However, due to the rarity of KTS and the lack of awareness among clinicians, a diagnosis of KTS was not made, and genetic testing was not completed at once. It is advisable to remain vigilant about the potential for KTS and complete genetic testing as soon as possible when there is a link between amelogenesis imperfecta, global developmental delay, and epilepsy.

Global developmental delay frequently causes significant distress for both KTS patients and their families. While most KTS cases exhibited global developmental delay or regression after the onset of epilepsy, approximately 20% of patients showed global retardation before epilepsy occurred [2–5, 11, 13, 14, 18]. According to the reported cases, such children usually start walking at 2–5 years of age [5, 8, 11, 13, 21], and some may lose the ability to walk during long-term follow-up due to spasms and abnormal gait [2, 13]. With a long period of uninterrupted rehabilitation training, our patient started walking when she was 25 months old. She could walk alone without spasms and abnormal gait for now, but his gait was not very stable yet (Supplementary material: Video 1). No genotype has been found to be associated with the degree of dyskinesia so far. Even patients with the same genotype can exhibit varying degrees of dyskinesia. For instance, two patients with the same mutation as in this study started walking independently at 24 and 21 months, respectively, which were similar to our patient [14]. However, one of the two patients was unable to stand up even at six years old. Therefore, we believe that this phenomenon is not only associated with the heterogeneity of clinical presentation but also with the persistence of rehabilitation training over six years.

Additionally, the language disabilities of KTS patients are significantly impacted. The *ROGDI* gene codes for a protein involved in the development of glial cells and neuron migration. Mutations in this gene may result in connectivity and communication issues between brain regions, which can affect both language development and comprehension [22, 23]. Table 1 presented a summary of our findings that indicated varying degrees of language impairment among all KTS patients for whom data was available. Language impairment manifests as either a complete absence of verbal communication or severely limited verbal abilities. The patient in our study also exhibited limited verbal communication skills.

There is currently no evidence that the frequency of seizures is associated with early-onset developmental delays in KTS patients [3, 5, 13, 18]. However, when it comes to epilepsy and developmental delays, epilepsy may lead to developmental delay, particularly with prolonged or frequent seizures. Conversely, developmental delay may increase the risk of epilepsy. Some genetic mutations, including *ROGDI* gene mutations, can result in the simultaneous occurrence of epilepsy and developmental delay. Hence, it is crucial to consider and manage both conditions concurrently in clinical management.

It is not clear whether KTS is associated with any comorbidities. However, our analysis of the limited number of cases available (Table 1) suggested that five patients had ADHD and one patient had suspected ASD [2, 8, 13, 18]. Although this evidence is not definitive, we speculate that there may be a link between KTS and comorbidities. Given that some KTS patients have exhibited self-harm behaviors, aggression, or impulsivity, it is strongly recommended to pay close attention to the possible presence of comorbidities [2, 11–13]. We advocate for strengthening psychological interventions and adopting multidisciplinary management approaches for KTS patients to prevent accidental injury.

Currently, no recommendations exist regarding the use of ASMs for these patients. However, we observed a positive response to PMP in the patient described in this report, which is consistent with a case reported by Lelde Liepina [12]. Following PMP treatment, the patient remained seizure-free for seven months and exhibited a significant reduction in epileptic discharges, as evidenced by VEEG results. Moreover, the patient’s mother reported that her child made notable improvements in gross motor development following PMP treatment. PMP is an anticonvulsant with a unique pharmacological profile. It acts as a non-competitive antagonist of AMPA receptors, which inhibits the AMPA receptor-mediated current in single neurons [24, 25]. The anticonvulsant effect of PMP is due to its ability to disrupt the AMPA receptor-dependent recruitment of pyramidal-inhibitory neuronal network oscillations. This disruption is achieved through dynamic glutamatergic and GABAergic transmission [26]. Given that *ROGDI* can influence GABA neurotransmission and the mechanism of PMP in treating epilepsy, we speculate that this may explain why PMP is effective in treating KTS patients [20]. The long-term effectiveness of PMP needs to be verified by longer follow-up. Nonetheless, the exact pharmacological mechanisms require additional investigation and clinical evidence. Regardless, we suggest that clinicians may contemplate PMP therapy for KTS patients with epilepsy as it could potentially decrease seizure frequency and enhance motor development.

## Conclusion

Our study reported the first case of *ROGDI*-related KTS in the Chinese population, which enriched the content of the spectrum disorder. We acknowledged the limitations of the single case report, as personalized treatment strategies must be developed based on the individual characteristics of each patient. Prospective multicenter clinical studies should be conducted to clarify the pathogenesis of KTS and the pharmacological effects of PMP treatment, which will assist clinical physicians in developing better disease management strategies.

### Supplementary Information

**Additional file 1: Supplementary material: Video 1. **The child's walking performance at age of six.

## Data Availability

The data that support the findings of this study are available from the corresponding author, Li Jiang, upon reasonable request. The datasets generated during the current study are available in the UNIPROT repository (https://www.uniprot.org/uniprotkb/Q14738/entry#sequences).

## Abbreviations

ADHD: Attention deficit hyperactivity disorder
ASD: Autism spectrum disorder
ASM: Anti-seizure medication
ACTH: Adrenocorticotropic hormone
CHCMU: Children’s Hospital of Chongqing Medical University
CLB: Clobazam
DQ: Developmental quotient
EEG: Electroencephalogram
GDS: Gesell Developmental Schedules
IDDs: Intellectual and developmental disabilities
KTS: Kohlschütter-Tönz syndrome
KD: Ketogenic diet
LEV: Levetiracetam
LOF: Loss of function
CHCMU: Medical University
MRI: Magnetic resonance imaging
NGS: Next-generation sequencing
PMP: Perampanel
PDMS: Peabody's developmental motor scale
PHB: Phenobarbital
RE: Refractory epilepsy
trio-WES: Trio whole exome sequencing
VEEG: Video electroencephalogram
VPA: Valproic acid
VNS: Vagus nerve stimulation
VGB: Vigabatrin
